# Valproic acid inhibits neural progenitor cell death by activation of NF-κB signaling pathway and up-regulation of Bcl-XL

**DOI:** 10.1186/1423-0127-18-48

**Published:** 2011-07-04

**Authors:** Hyo Sang Go, Jung Eun Seo, Ki Chan Kim, So Min Han, Pitna Kim, Young Sun Kang, Seol Heui Han, Chan Young Shin, Kwang Ho Ko

**Affiliations:** 1 Department of Pharmacology, College of Pharmacy, Seoul National University, Seoul, Korea; 2 Department of Neuroscience, School of Medicine and Center for Neuroscience Research, IBST, Konkuk University, Seoul, Korea; 3 Department of Biomedical Science & Technology, IBST, Konkuk University, Seoul, Korea

## Abstract

**Background:**

At the beginning of neurogenesis, massive brain cell death occurs and more than 50% of cells are eliminated by apoptosis along with neuronal differentiation. However, few studies were conducted so far regarding the regulation of neural progenitor cells (NPCs) death during development. Because of the physiological role of cell death during development, aberration of normal apoptotic cell death is detrimental to normal organogenesis.

Apoptosis occurs in not only neuron but also in NPCs and neuroblast. When growth and survival signals such as EGF or LIF are removed, apoptosis is activated as well as the induction of differentiation. To investigate the regulation of cell death during developmental stage, it is essential to investigate the regulation of apoptosis of NPCs.

**Methods:**

Neural progenitor cells were cultured from E14 embryonic brains of Sprague-Dawley rats. For *in vivo *VPA animal model, pregnant rats were treated with VPA (400 mg/kg S.C.) diluted with normal saline at E12. To analyze the cell death, we performed PI staining and PARP and caspase-3 cleavage assay. Expression level of proteins was investigated by Western blot and immunocytochemical assays. The level of mRNA expression was investigated by RT-PCR. Interaction of Bcl-XL gene promoter and NF-κB p65 was investigated by ChIP assay.

**Results:**

In this study, FACS analysis, PI staining and PARP and caspase-3 cleavage assay showed that VPA protects cultured NPCs from cell death after growth factor withdrawal both in basal and staurosporine- or hydrogen peroxide-stimulated conditions. The protective effect of prenatally injected VPA was also observed in E16 embryonic brain. Treatment of VPA decreased the level of IκBα and increased the nuclear translocation of NF-κB, which subsequently enhanced expression of anti-apoptotic protein Bcl-XL.

**Conclusion:**

To the best of our knowledge, this is the first report to indicate the reduced death of NPCs by VPA at developmentally critical periods through the degradation of IκBα and the activation of NF-κB signaling. The reduced NPCs death might underlie the neurodevelopmental defects collectively called fetal valproate syndrome, which shows symptoms such as mental retardation and autism-like behavior.

## Background

The importance of cell death for normal brain morphogenesis in the developing nervous system has been acknowledged since the beginning of modern neuroscience era [1,2]. Programmed cell death is a normal and physiological process to allow proper development of structure and function. In nervous system, more than half of the neurons die *via *apoptotic cell death during the developmental course [3-6].

At the level of the individual cell, apoptosis is triggered by a wide spectrum of stimuli during embryonic development, not only in response to stress and disease but also as a part of normal tissue homeostasis [3]. Because of the physiological role of cell death during development, aberration of normal apoptotic cell death is detrimental to normal organogenesis. Excessive cell death can result in functional deficits from the loss of specific cell populations, which occurs during age-associated neurodegenerative disorders [7,8], while reduced cell death may results in overgrowth and functional disorganization of the organ. In peripheral system, the loss of cells during development regulates sculpting digits, removing a tail, or eliminating lymphocytes of unwanted specificities [9]. Although the reduced neuronal death is not considered detrimental in vertebrates in the laboratory environment, reduced neuronal death *in vivo *may alter functional and structural properties of nervous systems leading to the developmental disorders with abnormal brain function.

Apoptosis occurs in not only neuron but also in NPCs and neuroblast [10-12]. When growth and survival signals such as EGF or LIF are removed, apoptosis is activated concomitant with the induction of differentiation [13,14]. To investigate the regulation of cell death during developmental stage, it is essential to investigate the regulation of apoptosis of NPCs.

Valproic acid (VPA), discovered as an anti-convulsant drug and is also used as the anti-bipolar disorder drug, regulates several signaling pathways in brain cells. VPA inhibits class I and II HDACs. *In vitro *studies showed that VPA specifically triggers phosphorylation of ERK, the upstream modulator of AP-1, without alteration of JNK and p38 pathways [15]. It also inhibits GSK-3β-mediated phosphorylation of proteins including β-catenin. In addition, VPA has been implicated in the regulation of LOX, PPARs, PTEN pathways [16]. Through the regulation of the above mentioned pathways, VPA has been generally considered to be neuroprotective.

Of interest is the recent reports suggesting the up-regulation of the cytoprotective protein Bcl-2 by VPA in neuron [17]. As we reported previously, VPA induces developmental defects when administered at developmentally critical periods [18], which includes functional deficits including mental retardation as well as structural abnormalities such as neural tube defects and the overgrowth of brain. In this study, we investigated whether VPA protects NPCs from cell death and if so, the mechanism by which VPA mediates the effects.

## Materials and methods

### Materials

Dulbecco's modified Eagle's medium/F12 (DMEM/F12), B27-serum free supplement and antibiotics were obtained from Gibco BRL (Gland Island, NY, USA). VPA, EGF, poly-L-ornithine and staurosporine were purchased from Sigma (St. Louis, MO). FGF was obtained from Invitrogen (Carlsbad, CA). H_2_O_2 _was purchased from Merck (Darmstadt, Germany). NF-κB inhibitor 4-Benzyl-2-methyl-1,2,4-thiadiazolidine-3,5-dione (TDZD-8) was obtained from Calbiochem-Novabiochem (San Diego, CA).

### Animals

Sprague Dawley (SD) rats were used throughout this study. Pregnant rats were injected with VPA or normal saline at E12 and brain tissues were dissected out from E14 and E16 embryos. Animal handling was in accordance with national guidelines and approved by the 'Seoul National University Institutional Animal Care and Use Committee (SNUIACUC)'.

### Neural progenitor cell culture

The preparation of cortical progenitors from embryos was based on the method previously described and slightly modified by us [19,20]. NPCs were prepared from E14 embryos of SD rats. Cortices were dissociated into single cells by mechanical trituration and the cells were incubated with Dulbecco's modified Eagle's medium/F12 (DMEM/F12) supplemented with B27-serum free supplement, 20 ng/ml EGF and 10 ng/ml FGF in a 5% CO_2 _incubator. EGF and FGF were added every day and the cells grew into floating neurospheres. The primary neurosphere was dissociated into single cells with trypsin-EDTA (GibcoBRL, a subsidiary of Invitrogen, Carlsbad, CA) and the cells were incubated as neurospheres in EGF and FGF containing media. This procedure was repeated and neurosphere colonies were again dissociated into single cells and plated on poly-L-ornithine coated plates with DMEM/F12 media containing 20 ng/ml EGF. The purity of culture was checked by immunostaining using an antibody against nestin, which is a marker for NPCs. In this study, 95% of cells were positive to nestin. Next day, the media was removed and NPCs were incubated with fresh growth factor-free media. One hour later, reagents were treated to NPCs culture. Protein samples were harvested 8 hours after VPA treatment for Western blot. Samples were fixed with 4% PFA 8 hours after VPA treatment for immunocytochemistry. For RT-PCR analysis, cellular RNA was harvested 2 hours after VPA treatment.

### Preparation of whole brain lysate

Whole brains were taken from embryonic day 14 and 16 animals. For the Western blot and RT-PCR, homogenized brain tissues were prepared in lysis buffer and Trizol respectively. Lysates were diluted by 2X sample buffer and adjusted to 1 μg/μl concentration of protein after the BCA protein assay.

### FACS analysis

NPCs dissociated into single cells were used to detect ratio of dead cells by FACS analysis. Approximately 1 × 10^6 ^cells were used for each analysis. NPCs were trypsinized and PBS containing 1% FBS was added. After PBS washing, cells were resuspended in PBS containing 1% FBS and 0.5 μg/ml propidium iodide (PI). The cell suspension was kept for 5 min at room temperature, and then ratios of positive or negative PI signal were measured by flow cytometry (FACS Calibur System, BD Biosciences, San Jose, CA). The experimental data were analyzed using CellQuest software.

### Western blot

NPCs were treated with VPA and 8 hours later, cells were washed with PBS and harvested with lysis buffer. Separation of protein bands was performed by 10 or 15% SDS-polyacrylamide gel electrophoresis and proteins were electrically transferred onto nitrocellulose (NC) membranes. The membranes were blocked with polyvinyl alcohol diluted in DDW (10 μg/ml). Membranes were then incubated overnight with the PARP-1 (1:5000, Santa Cruz, CA) and caspase-3 (1:5000, Cell Signaling, Danvers, MA) antibody, which were used as a marker for cell death. Other antibodies used in this study include NF-κB p65, NF-κB p50, Bcl-XL, Bax (1:5000, Santa Cruz, CA), IκBα (1:5000, Cell Signaling, Danvers, MA) and β-actin (1:10000, Sigma, St. Louis, MO). We used NUP98 (1:5000, Cell Signaling, Danvers, MA), which located at nuclear complex [21], as nuclear marker. As a cytoplasmic marker, α-tubulin (1:5000, Abcam, Cambridge, UK) was used. Membranes were washed 3 times with PBS-Tween (0.2% tween-20) for 10 min each, followed by incubation with horse-radish peroxidase-conjugated secondary antibody for 2 hours at room temperature. The membranes were developed with enhanced chemiluminescence (ECL) solution (Millipore, Billerica, MA) according to manufacturer's instructions. The signal intensities of blots were analyzed using 'Image J' (National Institutes of Health) software.

### Preparation of nuclear and cytoplasmic fractions

Nuclear extracts were prepared according to a method published previously [22]. Briefly, the cells in dishes were washed with PBS. Cells were then scraped, transferred to microtubes, and allowed to swell after the addition of 100 μl of hypotonic buffer containing 10 mM HEPES, pH 7.9, 10 mM KCl, 0.1 mM EDTA, 2 mM dithiothreitol (DTT), and 0.5 mM phenylmethylsulfonylfluoride. The lysates were incubated for 10 min in ice and centrifuged at 7,200 g for 5 min at 4°C. Supernatants were used as cytoplasmic fractions. After washing, pellets containing crude nuclei were resuspended in 50 μl of extraction buffer containing 20 mM HEPES, pH 7.9, 400 mM NaCl, 1 mM EDTA, 10 mM dithiothreitol, and 1 mM phenylmethylsulfonylfluoride and then incubated for 1 hour in ice. The samples were centrifuged at 12,000 g for 10 min to obtain supernatants containing nuclear fractions.

### The association of Bcl-xL promoter region with NF-κB: Chromatin immunoprecipitation (ChIP) analysis

ChIP analysis for the Bcl-xL promoter region was performed based on the method previously described [23]. NPCs were treated with VPA and 8 hours later, cells were washed with PBS. NPCs were prepared and cross-linked with 1% formaldehyde for 20 min at room temperature. Then formaldehyde was quenched with 125 mM glycine for 5 min at room temperature. Cells were scraped and collected by centrifugation (2,000 g for 5 min at 4°C), then washed twice with cold PBS. Pellets were resuspended with lysis buffer and centrifuged several times at 12,000 g for 1 min at 4°C and the supernatant was removed. The nuclear pellet was washed with 1 ml lysis buffer, by resuspending the pellet, followed by centrifugation. To shear the chromatin, the washed pellet was sonicated after resuspended in 1 ml of lysis buffer. The lysates were cleared by centrifuging at 12,000 g for 10 min at 4°C and the supernatants were retained. For IP, an antibody against NF-κB p65 was added to samples and the tube was rotated for 12 hours at 4°C. For mock IP, we incubated samples with beads without antibody. The precipitated chromatin was cleared by centrifugation at 12,000 g for 10 min at 4°C and the top 90% of cleared chromatin was transferred to a tube with protein A-agarose slurry and the tubes were rotated at 4°C for 45 min on a rotating platform. The slurry was washed at 2,000 g for few seconds and the supernatant was removed. The beads was washed 5 times with 1 ml cold lysis buffer then, 100 μl of 10% Chelex 100 slurry was directly added to the washed beads and boiled for 10 min. After centrifugation at 12,000 g for 1 min of 4°C, supernatants were transferred to a new tube. PCR amplification was carried out for 34 cycles, and PCR products were separated on 1.5% agarose gels. The PCR amplification was performed for 34 cycles (94°C, 0.5 min; 57°C, 0.5 min; 72°C, 1 min) with the following oligonucleotide primer sets and analyzed by DND gel electrophoresis:

For Bcl-XL

Forward primer: 5'-GGGAGTGGTCTTTCCGAA-3'

Reverse primer: 5'-CTCCATCGACCAGATCGA-3'

### RT-PCR

NPCs were treated with VPA and 2 hours later, cells were washed with PBS and total RNA was isolated from NPCs using Trizol reagent (Invitrogen) and 1 μg of total RNA was converted to cDNA using Superscript II reverse transcriptase according to the manufacturer's recommendation (Invitrogen). The PCR amplification was performed for 34 cycles (94°C, 0.5 min; 57°C, 0.5 min; 72°C, 1 min) with the following oligonucleotide primer sets:

For Bcl-XL

Forward primer: 5'-CCCCAGGACTTTGTACCTCA-3'

Reverse primer: 5'-TCCGAACGGTAAATGCCTAC-3'

For H,

Forward primer: 5'-TCCCTCAAGATTGTCAGCAA-3'

Reverse primer: 5'-AGATCCACAACGGATACATT-3'

### Immunocytochemistry (ICC)

NPCs were treated with VPA and 8 hours later, cultured NPCs on cover glasses (Fisher Scientific, Pittsburgh, PA) were washed and fixed with 4% paraformaldehyde at 37°C for 20 min. The cells were treated with 0.3% Triton X-100 for 10 min and blocked for 30 min with blocking buffer (1% BSA in PBS) at room temperature. The cells were incubated overnight at 4°C with primary antibodies against NF-κB p65 (1:500), IκBα (1:500), Bcl-XL (1:500), Bax (1:500), nestin (1:500, Millipore, Billerica, MA), and COX4 (a marker for mitochondria, 1:500, Cell Signaling, Danvers, MA) and washed with PBS for three times (0.1% BSA, 0.5% FBS in PBS). Secondary antibodies conjugated with either Rhodamine (1:500) or FITC (1:500), were diluted in blocking buffer and incubated for 2 h at room temperature. At this time DNA marker DAPI was also added to blocking buffer. After 3 washes with PBS, the cover glasses were mounted in mounting medium (Biomeda, Foster City, CA) and viewed with a fluorescence microscope. (Carl Zeiss, Germany).

### Statistical analysis

Data were expressed as the mean ± standard error of mean (S.E.M) and analyzed for statistical significance using one way analysis of variance (ANOVA) followed by Newman-Keuls test as a post hoc test and a P value < 0.05 was considered significant.

## Results

### VPA reduced NPCs cell death

**Figure 1:**
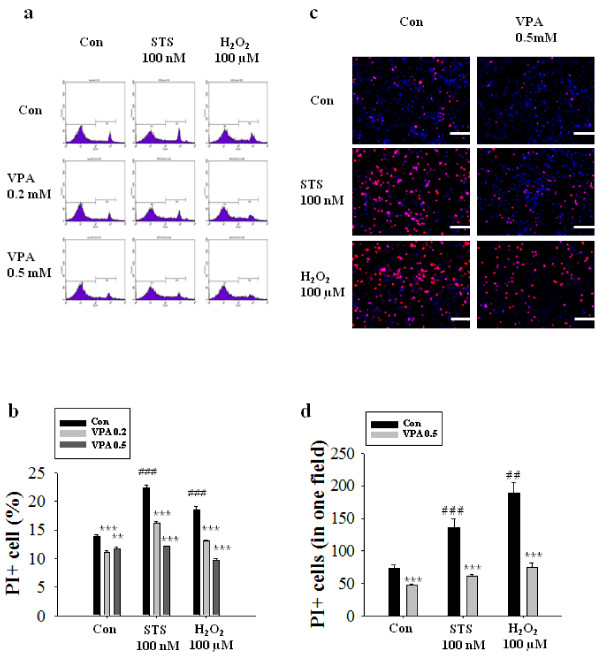
**Inhibition of basal, staurosporine- or hydrogen peroxide-induced cell death of NPCs by VPA**. NPCs culture were treated with 0.2 mM or 0.5 mM VPA or PBS (Vehicle) and stimulated with 100 nM staurosporine or 100 μM H_2_O_2_. Cells were analyzed by FACS analysis or PI staining 8 hr after the treatment as described in materials and methods. (A) The ratios of apoptotic cells were measured by FACS analysis after PI staining. (B) Quantification of FACS data (n = 3). (C) PI cytochemical staining. VPA decreased the number of PI positive cells. (D) Quantification of PI staining data (n = 6), Results are mean ± S.E.M. **P < 0.01 and ***P < 0.001 vs. Vehicle. ## p < 0.01, ### p < 0.001 vs. control. Scale bar represents 100 μm.

We first investigated whether VPA protects cultured NPCs from cell death. To induce cell death by withdrawing growth factors, we changed medium with fresh DMEM/F12 without growth factors. VPA (0.2 or 0.5 mM) was added at the time of media change. To induce stimulated cell death, 100 nM staurosporine or 100 μM H_2_O_2 _was also added to NPCs at 1 hour after VPA treatment in some cases. Cells were trypsinized and stained with PI solution 8 hr after media change. After growth factor deprivation, approximately 14% of NPCs showed shrinked morphology and was positive to PI. In FACS analysis, the ratio of PI positive dead cell was increased by staurosporine or H_2_O_2 _treatment. 0.2 and 0.5 mM of VPA decreased cell death in basal condition as well as staurosporine- or H_2_O_2 _-stimulated conditions (Figure 1A, B). To visualize the protective effect of VPA, we stained NPCs with PI solution, which gave similar results as FACS analysis (Figure 1C, D). In this condition, staurosporine and H_2_O_2 _didn't change either NF-κB expression or translocation to nucleus (data not shown).

### Valproic acid induced Bcl-XL expression via NF-κB signaling pathway

**Figure 2:**
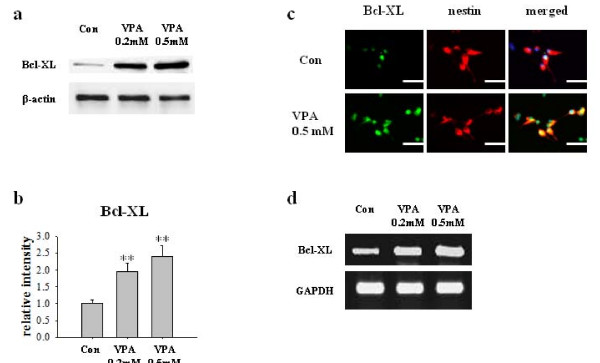
**Increased expression of Bcl-XL in NPCs by VPA**. (A) Bcl-XL expression was measured by Western blot after VPA treatment. VPA significantly increased Bcl-XL expression in a concentration dependent manner. Levels of β-actin were used as control, which was not different among groups. (B) Densitometric quantification of Bcl-XL expressions (n = 5). (C) Immunocytochemical staining of Bcl-XL expression in NPCs. Left, NPCs were stained with Bcl-XL. Middle, NPCs were stained with nestin, a marker of NPCs. Right, Bcl-XL, nestin, and DAPI stained fields were merged. (D) Bcl-XL mRNA expressions in NPCs were measured using RT-PCR. GAPDH was used as control. Results are mean ± S.E.M (n = 5). **P < 0.01 vs. control. Scale bar represents 50 μm.

**Figure 3:**
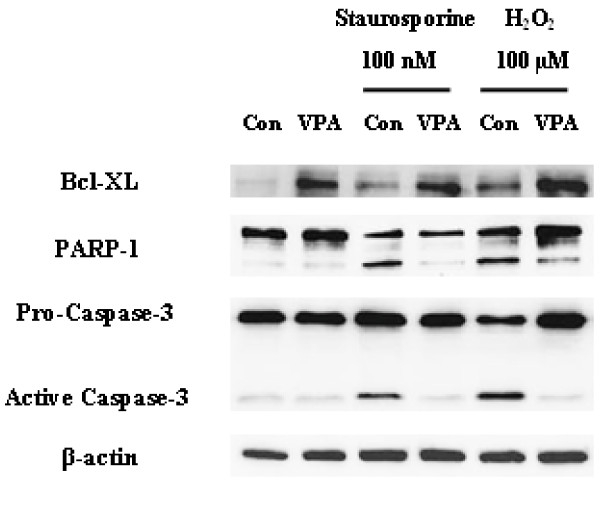
**VPA increased Bcl-XL expression and decreased PARP and caspase-3 cleavage in staurosporine- or H_2_O_2 _-stimulated NPCs**. The level of PARP-1, caspase-3 and Bcl-XL was determined by Western blot. VPA increased Bcl-XL expression and suppressed PARP-1 and caspase-3 cleavage induced by staurosporine or H_2_O_2 _in NPCs culture.

To determine the molecular mechanism of VPA-induced suppression of NPCs death, we investigate Bcl-XL and NF-κB signaling pathway. Bcl-XL is a well known anti-apoptotic molecule and highly expressed in NPCs as well as in brain during developmental period. We first investigated whether VPA changes the expression level of Bcl-XL in NPCs. VPA (0.2 mM and 0.5 mM) increased Bcl-XL protein and mRNA expression in a concentration-dependent manner in Western blot (Figure 2A, B), immunocytochemistry (Figure 2C) and RT-PCR (Figure 2D), respectively. VPA also increased Bcl-XL expression and inhibited PARP-1 cleavage and caspase-3 activation induced by staurosporine or H_2_O_2 _(Figure 3).

**Figure 4:**
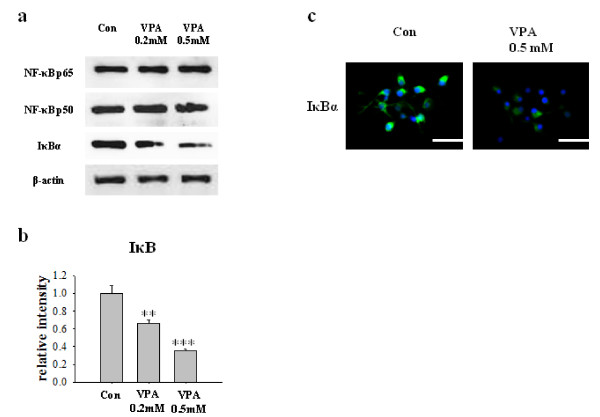
**VPA decreased the level of IκB**. (A) The level of NF-κB p65, NF-κB p50 and IκBα were measured by Western blot. (B) Densitometric quantification of the level of IκBα (n = 7). (C) Immunocytochemical staining of IκBα in NPCs. NPCs were stained with an antibody against IκBα and co-stained with DAPI. Results are mean ± S.E.M. **P < 0.01 and ***P < 0.001 vs. control. Scale bar represents 50 μm.

**Figure 5:**
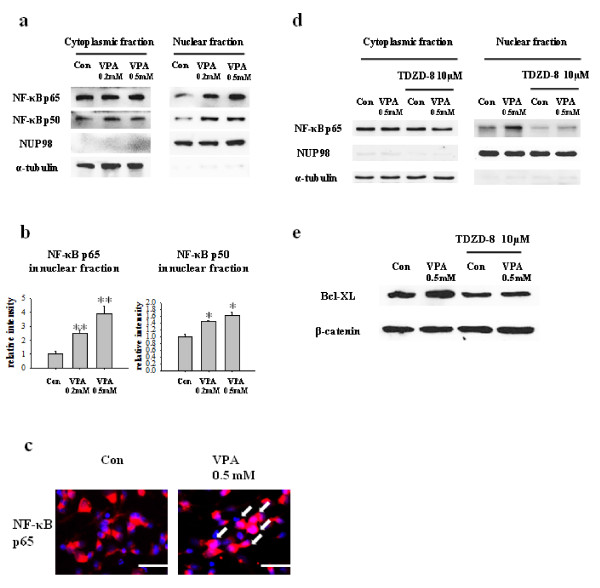
**VPA increased nuclear translocation of NF-κB**. (A) Nuclear translocation of NF-κB by VPA. NPCs cellular proteins were fractionated into nucleus and cytoplasmic fraction as described in materials and methods. The levels of NF-κB in each fraction were measured by Western blot. The level of NF-κB in nucleus was increased, whereas that in cytoplasm was not changed. (B) Densitometric quantification of NF-κB p65 and NF-κB p50 in nuclear fraction (n = 3). (C) Translocation of NF-κB p65 was confirmed by immuncytochemical staining. NPCs were co-stained with NF-κB p65 and DAPI. Arrows indicate nuclear localization of NF-κB p65. (D, E) An inhibitor of NF-κB, TDZD-8, was pre-treated 1 hour before VPA treatment and nuclear translocation of NF-κB p65 and Bcl-XL expression was determined as above (n = 3). Results are mean ± S.E.M. **P < 0.01 and ***P < 0.001 vs. control. Scale bar represents 50 μm.

**Figure 6:**
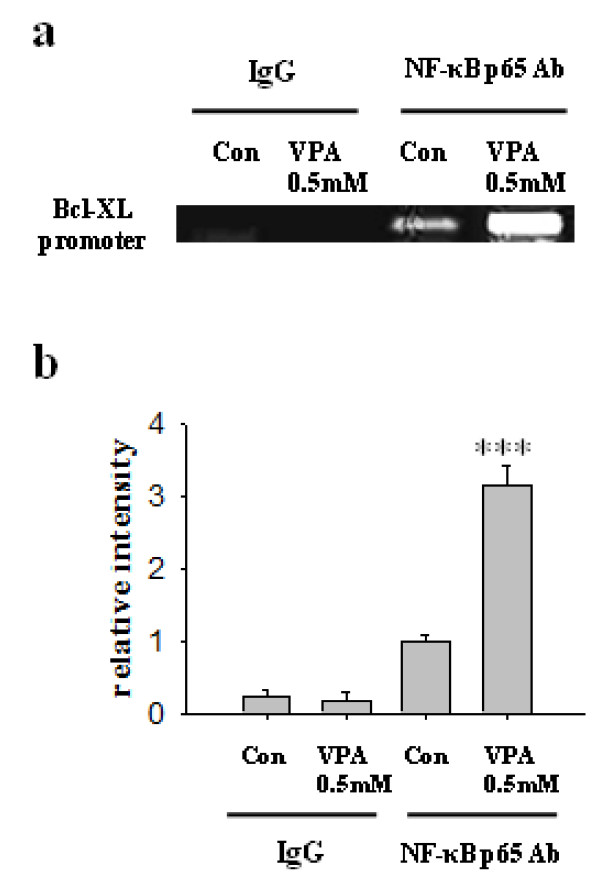
**Increased association of NF-κB with Bcl-XL gene promoter in response to VPA**. The association of NF-κB with Bcl-XL promoter was analyzed as described in materials and methods. (A) ChIP identifies the association of Bcl-XL promoter by NF-κB transcription factor. VPA significantly increased binding of NF-κB with Bcl-XL promoter region. (B) Densitometric quantification of ChIP assay (n = 3). Results are mean ± S.E.M. **P < 0.01 and ***P < 0.001 vs. control.

Next, we investigated the involvement of NF-κB signaling pathway. It was previously reported that NF-κB activation drives Bcl-XL promoter to increase the protein expression in hippocampal CA1 cells [24]. NF-κB pathway affects myriad of cellular responses including cell death and survival, which is mediated at least in part by the regulation of the expression of Bcl-XL. We hypothesized that VPA regulates NF-κB signaling pathway, subsequently increases expression of Bcl-XL. Although VPA did not affect the expression levels of NF-κB p65 and NF-κB p50, it decreased the level of IκBα, a biological inhibitor of NF-κB (Figure 4A, B). The decreased level of IκBα was also confirmed by immunocytochemistry (Figure 4C). To determine whether the decreased level of IκBα mediates the activation of NF-κB pathway, we investigated the nuclear translocation of NF-κB by performing Western blot of cytoplasmic and nuclear fraction of NPCs culture. Although the level of NF-κB in cytoplasmic fraction remains constant, NF-κB level in nuclear fraction was significantly increased. (Figure 5A, B) In addition, a lot of NF-κB immunoreactivity was localized in nucleus which was co-stained with DAPI in VPA group, whereas almost all the NF-κB immunoreactivity was localized in cytosplasm in control group (Figure 5C). Furthermore, 1 hour pretreatment of 10 μM of TDZD-8, a NF-κB inhibitor, suppressed VPA induced NF-κB p65nuclear translocation and Bcl-XL expression (Figure 5D, E). To unequivocally demonstrate the role of NF-κB pathway on VPA-induced Bcl-XL expression, we performed ChIP assay. VPA significantly increased interaction between NF-κB p65 and Bcl-XL promoter region (Figure 6A, B). These results suggest that VPA activates NF-κB by reducing the level of IκBα, which may up-regulate Bcl-XL expression to inhibit apoptosis of NPCs. Because VPA may triggers ERK phosphorylation [15], we tried to examine ERK activation by VPA, however, we did not observe consistent increase in ERK activation by VPA (data not shown).

### VPA induces Bcl-XL expression in developing rat brain

**Figure 7:**
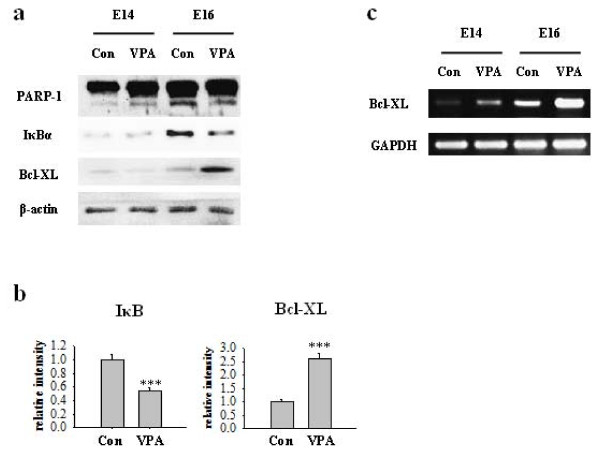
**VPA up-regulates Bcl-XL expression and inhibits cell death *in vivo***. VPA was injected into pregnant rats at E12 and embryonic whole brain lysates of control and VPA group at each embryonic day (E14 and E16) were obtained as described in materials and methods. (A) Western blot of apoptotic markers PARP-1 cleavage was reduced in VPA model at E16. IκBα level was decreased and Bcl-XL expression was increased in VPA group at E16. (B) Densitometric quantification of IκBα (n = 5) and Bcl-XL. (n = 7) (C) Bcl-XL mRNA level was measure by RT-PCR, which was increased at E14 and E16. Results are mean ± S.E.M. ***P < 0.001 vs. control.

We injected 400 mg/kg of VPA or normal saline to pregnant rat at E12 to investigate whether VPA inhibits cell death *in vivo*. Although the level of PARP-1 cleavage in embryonic cortex was not significantly changed at E14, it was decreased at E16 by VPA injection. Similar to *in vitro *results, the level of IκBα was markedly decreased, whereas that of Bcl-XL was significantly increased at E16 (Figure 7A, B). The expression of Bcl-XL mRNA was also increased in E14 and E16 brain of VPA injected rats (Figure 7C). The effect of single injection of VPA was not sustainable and no other definite changes of PARP-1 cleavage, IκBα or Bcl-XL were detected at E18 and postnatal day 2 (data not shown). These results suggest that prenatal exposure to VPA may reduce the physiological apoptotic cell death of NPCs *in vivo *by mechanism involving degradation of IκBα and overexpression of Bcl-XL.

### VPA suppressed Bax expression in cultured NPCs and developing rat brain

**Figure 8:**
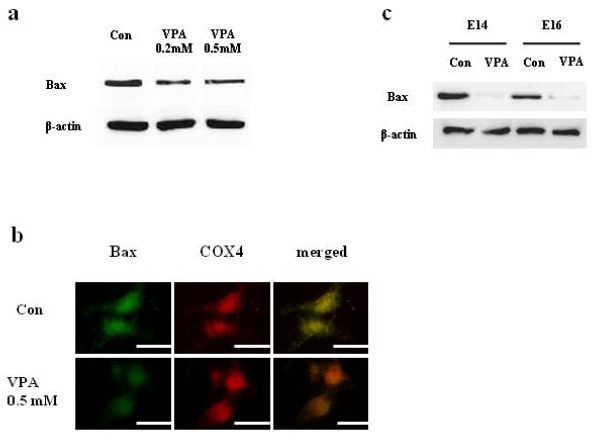
**VPA suppressed Bax expression both *in vitro *and *in vivo***. (A) Bax expression was measured by Western blot. VPA reduced Bax expression in cultured NPCs. (B) The reduction of Bax expression was confirmed by immunocytochemical staining. Left, NPCs were stained with Bax. Middle, NPCs were stained with COX4, a marker of mitochondria. Right, merged image of Bax and COX4 staining. (C) VPA was injected into pregnant rats at E12 and Bax level in embryonic brain was determined at E14 and E16. The level of Bax in brain was decreased at both time points. Scale bar represents 10 μm.

VPA regulated not only anti-apoptotic Bcl-XL expression, but also down-regulated pro-apoptotic Bax expressions both in cultured NPCs (Figure 8A, B) and in developing rat brains (E14 and E16, Figure 8C), which inhibits Bcl-XL by hetero-dimerizing with Bcl-XL [25]. Double immunostaining result of NPCs with antibodies against Bax and COX4, a marker of mitochondria, showed that the expression of Bax in COX4 positive mitochondria was also decreased as was the case in cytosol. The up-regulation of Bcl-XL with concomitant down-regulation of Bax by VPA may facilitate the inhibition of apoptotic cell death of NPCs.

## Discussion

### Activation of NF-κB signaling pathway by VPA

In this study, down-regulation of IκBα by VPA mediated the activation of NF-κB as evidenced by increased translocation of p65 and p50 to nucleus. In the canonical NF-κB activation pathway, an IKK complex composed of IKKα, IKKβ, and IKKγ phosphorylates IκB, leading to its degradation and the activation of NF-κB [26]. Many studies have reported evidences directly implicating the involvement of NF-κB/Rel family of transcription factors in the control of apoptosis in the immune and nervous systems. For example, NF-κB p65-/- double knockout mice demonstrated embryonic lethality because of massive apoptosis [27].

Growing number of studies reported that NF-κB activation is not only involved in the nervous system response to injury or inflammation, but also in neuronal survival in developing brain as well as in the adult nervous system. Although many studies suggested that VPA down-regulates NF-κB activity possibly *via *increase in acetylation on NF-κB, which may contribute to the anti-inflammatory effects of VPA in nervous system [28-30], at least one study suggested that VPA protects neuron from oxidative stress-induced cell death by acetylation-induced activation of NF-κB, although it's still possible that inhibition of JNK activity mediates the observed protective effects of VPA [31]. Similar pro-survival role of VPA has been reported in hippocampal NPCs [32]. In addition to above reports, we provide here new mechanism for the regulation of NF-κB pathway by VPA, i.e. the down-regulation of IκB. The different effects of VPA on the regulation of NF-κB activity in different experimental conditions suggest that the regulatory effects of VPA may differ in the context of dosing and time window of treatment in different cell types.

At present, the mechanism by which VPA down-regulates IκB is not clear. Although the transcriptional or translational down-regulation of IκB is also possible, one interesting hypothesis is the rapid degradation of IκB by VPA, especially considering the rapid degradation of IκB by proteasomal pathway (for a review, see [33]). In fact, VPA induced the proteasomal degradation of HDAC2, which might be regulated by the induction of E2 ubiquitin conjugase Ubc8 and increased ubuiquitination [34]. The concentration of VPA (0.2-0.5 mM) used in this study is similar with the concentration of VPA required for the reduction of HDAC2 protein levels as well as that required for inhibition of HDAC enzymatic activity. In our experiment, we also observed that 0.2 or 0.5 mM VPA strongly increased histone acetylation in NPCs, which may suggest that the concentration of VPA used in this study might be in the range of HDAC inhibitory concentration as well as that required for the regulation of ubiquitination-dependent HDAC degradation. In other study, VPA decreased steroid secretion by increasing the ubiquitination and degradation of SF-1 in similar dose ranges [35]. Although the ubiquitination dependent regulation of key regulatory molecules in NPCs by VPA is an emerging area of investigation, which we hope to explore further using a series of biochemical and molecular biological tools in the future, these results suggest that VPA might regulate the ubiquitination and degradation of signaling molecules in a clinically relevant concentration r. Alternatively, VPA may induce the phosphorylation of IκB for its degradation through the activation of PI3K-Akt-GSK3β pathways or ERK 1/2 pathways, the two well known target pathways regulated by VPA. Those two possibilities are now under active investigation in this laboratory.

### Regulation of the expression of anti-apoptotic protein Bcl-XL by VPA

Although Bcl-2 is the prototype of anti-apoptotic protein and is extensively studied so far, Bcl-2 levels decline rapidly during development [36] and targeted disruption of Bcl-2 resulted in only subtle neurodevelopmental abnormalities [37]. These observations suggest that other Bcl-2 family members may play a more significant role in the development of embryonic brain. Bcl-XL is another anti-apoptotic Bcl-2 family member which is expressed at relatively high levels in the nervous system [38,39]. In contrast to Bcl-2, high levels of Bcl-XL are maintained throughout development into adulthood [36,38-40] and the Bcl-XL-/- mice die during embryonic period [36] with extensive apoptotic cell death in the developing nervous system [41]. In our experiment, VPA up-regulated Bcl-XL expression and inhibited NPCs cell death in basal condition as well as pro-apoptotic condition induced by staurosporine and H_2_O_2_.

### VPA induced abnormality in cell death during development may implicates the underlying mechanism of hyperneurogenesis in some developmental disorders

In our study, VPA inhibited apoptotic cell death of NPCs. The decreased apoptosis may contribute to the increased NPCs resulting in hyper-differentiation of neuron in VPA-treated subjects. VPA is a potent teratogen and causes behavioral and neuroanatomical abnormalities similar to those seen in autism [42-45]. Interestingly, one of the anatomical feature observed in autism patients is macrocephaly and increased neuron density [46,47]. Prenatal VPA exposure model is one of the most widely used animal model for autism [43]. These results suggest that exposure to VPA during developmentally critical periods may contribute to the anatomical abnormalities similar to autism, possibly via increased NF-κB activation and decreased apoptotic cell death. Although it is also possible that increased proliferation of NPCs may lead to increased neuronal density, which is under active investigation in our lab, the involvement of similar mechanism, i.e. decreased apoptotic cell death, in other neurodevelopmental disorders such as tuberous sclerosis and Cowden syndrome would be an intriguing topic to be resolved in the future study.

One of the important issues in this study is the clinical relevance of the concentration of VPA used in this study. In many studies, 0.2 - 2 mM of VPA was generally used in neuron [48] as well as neural stem cell [49], which is higher than the VPA concentration used in this study (0.2-0.5 mM) without any immediate toxicity [50,51]. The concentration of VPA used in our in vitro study (0.2 mM or 0.5 mM) corresponds to 28.8 μg/mL or 72.1 μg/mL, which is slightly higher but well within the clinical concentration of VPA. In an animal model of autism, 400 mg/kg or 600 mg/kg of VPA was routinely used to mimic the human VPA exposure during pregnancy [43]. Serum or plasma VPA concentrations are generally in a range of 20-100 μg/mL during controlled therapy against epilepsy or bipolar disorder, with free concentration of VPA in blood and brain ranging from 7% to 28% of the total levels. In rats treated with 400 mg/kg VPA may have blood VPA concentration of 30-50 μg/mL [52] and the concentration of VPA in brain was one fifth of blood concentration [53]. In this regard, VPA concentration of brain in 400 mg/kg VPA injected animal may be calculated to approximately 6-10 μg/mL, similar to clinical VPA concentration of human brain (7.5 - 20.8 μg/mL).

## Conclusion

In this study, we provided evidences suggesting that VPA can suppress cell death of NPCs *via *regulation of NF-κB pathway and Bcl-XL expression. Prenatal exposure to valproic acid produces neurodevelopmental and somatic abnormalities collectively called fetal valproate syndrome, which includes the behavioral and anatomical symptoms similar to those seen in autism [42,45]. Defining the role of the diminished apoptotic cell death by exposure to VPA during developmentally critical period on the manifestation of the anatomical and behavioral defects would provide more insights into the pathogenesis of the neurodevelopmental disorders.

## Competing interests

The authors declare that they have no competing interests.

## Authors' contributions

HSG participated in study design and conceptualization, analyzed data, and wrote the manuscript. JES participated in study design and performed experiment. KCK helped with composing manuscript. SMH performed experiment. PK helped with experiment. SHH and YSK participated in study design. CYS conceptualized and designed the study and wrote the manuscript. KHK contributed study design and revised the manuscript for intellectual content. All authors read and approved the final manuscript.
